# Surveillance of SARS-CoV-2 transmission in educational institutions, August to December 2020, Germany

**DOI:** 10.1017/S0950268821002077

**Published:** 2021-09-22

**Authors:** Anja Schoeps, Dietmar Hoffmann, Claudia Tamm, Bianca Vollmer, Sabine Haag, Tina Kaffenberger, Kimberly Ferguson-Beiser, Berit Kohlhase-Griebel, Silke Basenach, Andrea Missal, Katja Höfling, Harald Michels, Anett Schall, Holger Kappes, Manfred Vogt, Klaus Jahn, Till Bärnighausen, Philipp Zanger

**Affiliations:** 1 Federal State Agency for Consumer & Health Protection Rhineland-Palatinate, Koblenz, Germany; 2 Heidelberg Institute of Global Heath, University Hospitals, Im Neuenheimer Feld 130.3, 69120 Heidelberg, Germany; 3 District Public Health Authority, Große Langgasse 29, 55116 Mainz, Germany; 4 District Public Health Authority, Peter-Altmaier Platz 1, 56410 Montabaur, Germany; 5 District Public Health Authority, Dörrhorststraße 36, 67059 Ludwigshafen, Germany; 6 District Public Health Authority, Ernst-Ludwig-Straße 36, 55232 Alzey, Germany; 7 District Public Health Authority, Neumayerstraße 10, 67433 Neustadt, Germany; 8 District Public Health Authority, Trierer Straße 49-51, 66869 Kusel, Germany; 9 District Public Health Authority, In der Malzdürre 7, 57610 Altenkirchen, Germany; 10 District Public Health Authority, Paulinstraße 60, 54292 Trier, Germany; 11 District Public Health Authority, Arzheimer Str. 1, 76829 Landau in der Pfalz, Germany; 12 District Public Health Authority, Trierer Straße 1, 54634 Bitburg, Germany; 13 Ministry of Health, Federal State of Rhineland-Palatinate, Bauhofstraße 9, 55116 Mainz, Germany; 14 Harvard Center for Population and Development Studies, Harvard University, Cambridge, USA; 15 Department of Global Health and Population, Harvard School of Public Health, Boston, USA; 16 Department of Infectious Diseases, Medical Microbiology and Hygiene, University Hospitals, Im Neuenheimer Feld 324, 69120 Heidelberg, Germany

**Keywords:** COVID-19, daycare, infectious disease epidemiology, schools, transmission

## Abstract

This study aims at providing estimates on the transmission risk of SARS-CoV-2 in schools and day-care centres. We calculated secondary attack rates (SARs) using individual-level data from state-wide mandatory notification of index cases in educational institutions, followed by contact tracing and PCR-testing of high-risk contacts. From August to December 2020, every sixth of overall 784 independent index cases was associated with secondary cases in educational institutions. Monitoring of 14 594 institutional high-risk contacts (89% PCR-tested) of 441 index cases during quarantine revealed 196 secondary cases (SAR 1.34%, 0.99–1.78). SARS-CoV-2 infection among high-risk contacts was more likely around teacher-indexes compared to student-/child-indexes (incidence rate ratio (IRR) 3.17, 1.79–5.59), and in day-care centres compared to secondary schools (IRR 3.23, 1.76–5.91), mainly due to clusters around teacher-indexes in day-care containing a higher mean number of secondary cases per index case (142/113 = 1.26) than clusters around student-indexes in schools (82/474 = 0.17). In 2020, SARS-CoV-2 transmission risk in educational settings was low overall, but varied strongly between setting and role of the index case, indicating the chance for targeted intervention. Surveillance of SARS-CoV-2 transmission in educational institutions can powerfully inform public health policy and improve educational justice during the pandemic.

## Introduction

The SARS-CoV-2 pandemic urges government leaders to define priorities when implementing anti-epidemic measures in public domains. This task requires a profound scientific basis when balancing the obvious benefits of anti-epidemic measures against their hazards on the social, economic and health sector. While the educational and psychological impact of school closures on child health is rather well studied [1–3], our knowledge on COVID-19 transmission risk in educational institutions is insufficient to adequately balance the risks and benefits of their closure: A number of smaller cohort studies from mid-2020 studied transmission risk in overall 171 index cases and their 6910 contact persons in Australian, Italian, Irish, Singaporean and German schools and report attack rates between 0% and 3% [4–8]. Based on data from the first wave of COVID-19, a number of modelling studies provide inconclusive guidance to policy makers. While two publications, one from several countries and one from Switzerland [9, 10], concluded that school closures contributed markedly to the reduction of SARS-CoV-2 transmission and individual mobility, respectively, two other studies, one using cross-country data and one from Japan rated school closures among the least effective measures to reduce COVID-19 incidence rates [11, 12]. Accordingly, a recent review on SARS-CoV-2 setting-specific transmission rates concluded that there is ‘limited data to explore transmission patterns in […] schools […], highlighting the need for further research in such settings’ [13].

The present study provides estimates of the SARS-CoV-2 transmission risk in schools and day-care centres during exponentially increasing COVID-19 population incidence from August to December 2020 in Germany, i.e. before the advent of SARS-CoV-2 variants of concern. Due to the large sample size, we also present analyses on the variation in transmission risk by a variety of contextual characteristics of the index case.

## Methods

### Source population

The presented data were collected in Rhineland-Palatinate, one of the 16 Federal States of Germany with an overall population of about 4.1 million, 1492 schools, 406 607 school-children and 144 245 children below 6 years of age in day-care [14, 15]. We report observations from the re-opening of educational institutions after the summer break, on 17 August, to their closure for a hard lock-down, on 16 December 2020. During this period, schools and day-care centres remained open throughout, with full face-to-face attendance, and the following publicly recommended hygiene measures in place: in secondary schools (i.e. age 10 years and older) (i) physical distancing (>1.5 m, except during classes), (ii) cross- or pulse-ventilation of class-rooms before and after class, and then every 20 min for 5 min during class [16], (iii) face masks in school-buildings and ‘on campus’, but not in the class-room, (iv) increased frequency of surface cleaning, and (v) structural support of individual hygiene (hand, cough etiquette) [17, 18]. On 2 November 2020, this concept was modified by additionally recommending face masks inside the classroom [18]. Comparable recommendations existed for primary schools and day-care centres, with the exception that in those institutions both, children and teachers, were exempted from physical distancing and wearing of face masks [19].

### Origin of index cases and contact persons

The 24 District Public Health Authorities (DPHAs) in Rhineland-Palatinate are responsible for all investigations around notifiable diseases, and represent populations between 61 000 and 430 000 individuals per district. Following the identification and statutory notification of a COVID-19 case, qualified personnel at the competent DPHA interviews this index case, tries to investigate the source of infection (primary case), traces contacts, and initiates a quarantine and active follow-up in those at high risk of transmission (category-I contact) for 14 days after the last contact to the index case. A category-I contact was defined as a person who either stayed face-to-face (<1.5 m) with a COVID-19 case for 15 min or longer, or in the same room (i.e. irrespective of distance) for 30 min or longer [20]. According to German guidelines, free PCR-testing is offered by the DPHAs to all category-I contact persons, irrespective of their symptom status [20]. Depending on the DPHAs organizational structure, the testing is organized by the DPHA personnel or by external structures, such as community testing centres. In the latter case, only SARS-CoV-2-positive PCR tests of secondary cases are notified to the DPHA, with the result that negative test results are only available to the DPHAs who organize testing themselves. This explains why DPHAs with external testing have (due to potentially incomplete uptake) missing information on the total number of tests done in contact persons of a given index case, even when such testing was routinely offered to all contact persons as a standard procedure for all index cases included in this study.

### Study setting and definitions

At the start of the study, a two-page data form was distributed to all DPHAs, alongside with instructions on inclusion and exclusion criteria for index cases and a link to upload completed data forms (ec.europa.eu/eusurvey). For inclusion into this study, an index case was defined as an individual that (i) tested positive for SARS-CoV-2-RNA from respiratory material, (ii) was notified as working in or attending an educational institution, and (iii) had worked in or attended the institution for at least one day during the infectious period. The infectious period was defined as follows: (i) for symptomatic index cases as time from 2 days before to 10 days after onset of symptoms; (ii) for asymptomatic cases with unknown origin from 2 days before to 10 days after the date of taking the diagnostic swab; and (iii) for asymptomatic cases with known contact to a primary case from 3 days to 15 days after exposure.

A secondary case was defined as an individual that (i) was identified as a category-I contact person to an index case by the competent DPHA, (ii) tested positive for SARS-CoV-2-RNA during the quarantine associated with that index case, and (iii) was unlikely a co-primary case, based on evidence on the assumed chain of infection that evolved during the competent DPHA's investigation. Contact persons and secondary cases not attending the educational institution, e.g. persons living in the same household, were not to be reported in this data form. Beginning with 17 August 2020, we asked DPHAs to file one data form for each eligible index case about 2 weeks after its identification, when information on all potential secondary cases would be available, i.e. after completion of quarantine of the contact persons.

### Statistical analysis

Assuming equal exposure time in all contact persons of a given index case, we calculated secondary attack rates (SARs, i.e. individual-level risk of transmission) as the proportion of secondary cases among high-risk contact persons of a given index case during a 14-day period following the last contact (i.e. quarantine), together with corresponding binomial 95% confidence intervals (95% CIs), taking account of the clustering of contact persons in index cases [21]. Associations between transmission risk on the individual level and a number of the index case's characteristics were analysed using negative binomial regression, providing estimates of the crude incidence rate ratio (IRR) comparing the SAR in exposed and unexposed together with its 95% CI and a *P*-value testing H_0_: ‘both SARs are equal’ (IRR = 1.0). For sensitivity, we repeated these analyses based on the number of PCR-tested contacts only.

The risk of causing a SARS-CoV-2 cluster (in brief ‘cluster risk’) was calculated by dividing the number of index cases that caused one or more secondary cases by the total number of observed index cases. To compare cluster risks by exposure category, we estimated risk ratios (RRs) and their 95% CIs and *P*-values testing H_0_: RR = 1.0 using binomial regression. Since this part of the analysis did not rely on an individual contact person denominator, it also included data from index cases with missing information on the total number of contact persons and/or the number of PCR tests done.

Finally, we compared the mean number of secondary cases per index (MSPI) among children/students and teachers, associated with index cases in students/children and in teachers in different institutions, respectively. As with the SARs, we used negative binomial regression models to compare differences in the count of secondary cases per index case by exposure category. All analyses and models accounted for clustering of secondary cases by index case and were conducted using Stata SE version 16.1 and SAS version 9.4 [22, 23].

### Ethical statement

The collection, analysis and communication of the presented data take place in response to the global COVID-19 pandemic and are mandated by the German Infectious Diseases Protection Act. Ethical approval was waived by the competent ethics committee, Federal State Medical Council (Landesärztekammer) in Rhineland-Palatinate, Mainz, Germany (application no. 2021-15634-r).

## Results

### Source population

**Fig. 1.**
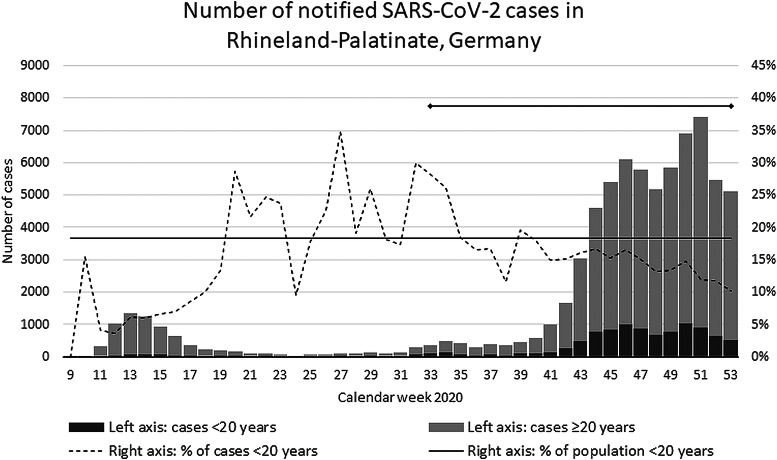
Epidemic curve of notified SARS-CoV-2 cases in children and adolescents, Rhineland-Palatinate, Germany, 2020. Figure displays the number (%) of SARS-CoV-2 cases notified in the Federal State of Rhineland-Palatinate (~4 million population) by calendar week, overall and among subjects < and ≥20 years of age.

The course of the COVID-19 pandemic in Rhineland-Palatinate was comparable to all of Germany and was characterized by an exponential increase from the end of September (calendar week 39) until the end of October (cw43), a further growth at a lower rate (cw44–45), followed by a fluctuation on a high level of about 5000–7000 new cases per week (cw46–52), which equals a 7-day incidence rate of 120–170 per 100 000 (Fig. 1). Sixteen per cent of the 74 733 COVID-19 cases notified in Rhineland-Palatinate in 2020 were younger than 20 years, which approximates the population proportion of 18.3% in this age group. By the end of the study period, SARS-CoV-2 Variant of Concern (VoC) alpha (i.e. ‘British Variant’) had been detected in overall six out of the overall 60 405 cases notified by 16 December in Rhineland-Palatinate, Germany. No other VoC was present in Germany at that time.

### Risk of SARS-CoV-2 transmission

Overall, DPHAs provided information for a total of 784 independent notified index cases attending an educational institution prior to diagnosis of a SARS-CoV-2 infection (591 students/children, 157 teachers, 36 unknown roles). There were 130 clusters of secondary cases reported via the SARS-Surveillance (cluster risk 0.17, 95% CI 0.14–0.19) (Table 1, right). Full information on PCR-testing (i.e. on positive and negative results) was available for 14 591 contact persons to 441 index cases (median 25 contacts per index case, IQR 17–40). Among these, the DPHAs identified 81 clusters with 196 PCR-positive secondary cases (SAR 1.34%, 95% CI 0.99–1.78%) (Table 1, left). Repeating the analysis based on only the 13 005 PCR-tested contacts (PCR-coverage 89%) gave an overall SAR of 1.51 (95% CI 1.11–2.00). The majority of contacts (74%) were tested between 7 and 10 days after their last contact with the index case (Supplementary Fig. S1).

**Table 1.** Risk of SARS-CoV-2 transmission in educational settings, by the characteristic of the index case, Germany, 2020

Characteristic of index case	Index cases^a^	Incident SARS-CoV-2 cases among 14 591 exposed high-risk contacts, during 14 days of follow-up (quarantine) *N* = 441 cohorts						Index cases^b^	Incident SARS-CoV-2 clusters among high-risk contacts, during 14 days of follow-up (quarantine) *N* = 784 cohorts				
		Secondary cases (clusters)	No. of contacts	% Contacts with PCR	SAR (%) [95% CI]	Crude IRR [95% CI]	*P*-value		Clusters (secondary cases)	Mean cluster size	Cluster risk [95% CI]	Crude RR [95% CI]	*P*-value
Role													
Student/child	346	99 (53)	10 716	87.47	0.92 [0.64–1.29]	1.00	ref	591	76 (145)	1.9	0.13 [0.10–0.16]	1.00	ref
Teacher	75	91 (25)	2858	92.93	3.18 [1.95–4.89]	3.17 [1.79–5.59]	<0.001	157	48 (169)	3.5	0.31 [0.23–0.38]	2.38 [1.73–3.26]	<0.001
unknown	20	6 (3)	1017	95.97	0.59 [0.09–1.93]	0.77 [0.23–2.63]	0.68	36	6 (15)	2.5	0.17 [0.06–0.33]	1.30 [0.61–2.77]	0.50
Symptom status													
Symptomatic	300	166 (64)	10 566	88.11	1.57 [1.12–2.14]	1.00	ref	550	100 (262)	2.6	0.18 [0.15–0.22]	1.00	ref
Asymptomatic	127	21 (13)	3523	91.26	0.60 [0.25–1.19]	0.47 [0.25–0.89]	0.02	203	24 (55)	2.3	0.12 [0.08–0.17]	0.65 [0.43–0.99]	0.04
Unknown	14	9 (4)	502	95.62	1.79 [0.43–4.84]	1.34 [0.37–4.85]	0.65	31	6 (12)	2.0	0.19 [0.07–0.37]	1.06 [0.51–2.23]	0.87
Type of institution^c^													
Day-care (0–6 years)	99	110 (32)	4392	90.64	2.50 [1.60–3.72]	3.23 [1.76–5.91]	<0.001	205	61 (203)	3.3	0.30 [0.24–0.37]	2.78 [1.88–4.10]	<0.001
Primary schools	88	27 (15)	2389	85.64	1.13 [0.57–1.99]	1.62 [0.80–3.31]	0.18	157	21 (40)	1.9	0.13 [0.08–0.20]	1.25 [0.75–2.09]	0.40
Secondary schools	173	41 (25)	5970	90.08	0.69 [0.39–1.12]	1.00	ref	299	32 (50)	1.6	0.11 [0.07–0.15]	1.00	ref
Vocational schools	52	5 (4)	1181	84.17	0.42 [0.10–1.14]	0.65 [0.21–1.99]	0.45	70	8 (14)	1.8	0.11 [0.05–0.21]	1.07 [0.51–2.22]	0.18
Other/unknown	29	13 (5)	659	91.96	1.97 [0.44–5.46]	2.99 [1.12–7.98]	0.03	53	8 (22)	2.8	0.15 [0.07–0.28]	1.41 [0.69–2.89]	0.35
Age													
0–5 years	42	31 (11)	1828	90.43	1.70 [0.79–3.18]	1.29 [0.55–3.03]	0.55	77	18 (45)	2.5	0.23 [0.14–0.34]	2.00 [1.14–3.53]	0.02
6–10 years	89	15 (12)	2410	82.53	0.62 [0.29–1.17]	0.72 [0.32–1.63]	0.43	155	18 (31)	1.7	0.12 [0.07–0.18]	1.00 [0.56–1.79]	0.99
11–15 years	113	35 (20)	3358	89.93	1.04 [0.55–1.80]	1.00	ref	189	22 (38)	1.7	0.12 [0.07–0.17]	1.00	ref
16–20 years	90	17 (9)	2884	88.04	0.59 [0.21–1.30]	0.56 [0.25–1.24]	0.15	157	16 (37)	2.3	0.10 [0.06–0.16]	0.88 [0.48–1.61]	0.67
21–34 years	42	30 (13)	1321	88.19	2.27 [1.02–4.33]	1.97 [0.84–4.64]	0.12	80	23 (50)	2.2	0.29 [0.19–0.40]	2.47 [1.46–4.17]	0.001
35 years and older	45	62 (13)	1773	93.80	3.50 [1.80–6.07]	2.80 [1.27–6.20]	0.01	90	27 (113)	4.2	0.30 [0.21–0.41]	2.58 [1.56–4.27]	<0.001
unknown	20	6 (3)	1017	95.97	0.59 [0.09–1.93]	0.65 [0.18–2.35]	0.51	36	6 (15)	2.5	0.17 [0.06–0.33]	1.43 [0.62–3.28]	0.40
Type of groups													
Stable groups	313	80 (52)	8650	88.15	0.92 [0.66–1.26]	1.00	ref	536	75 (123)	1.6	0.14 [0.11–0.17]	1.00	ref
Dynamic groups	111	91 (25)	5430	90.90	1.68 [1.04–2.56]	1.45 [0.84–2.50]	0.18	199	44 (168)	3.8	0.22 [0.17–0.29]	1.58 [1.13–2.21]	0.007
Unknown	17	25 (4)	511	86.89	4.89 [1.16–12.91]	3.21 [1.07–9.59]	0.04	49	11 (38)	3.5	0.22 [0.12–0.37]	1.60 [0.92–2.81].	0.10
Sex													
Female	194	66 (33)	5808	88.86	1.14 [0.70–1.73]	1.00	ref	332	43 (89)	2.1	0.13 [0.10–0.17]	1.00	ref
Male	241	128 (47)	8555	89.32	1.50 [0.99–2.16]	0.78 [0.46–1.31]	0.35	441	86 (238)	2.8	0.20 [0.16–0.24]	0.66 [0.47–0.93]	0.02
Unknown	6	2 (1)	228	89.04	0.88 [0.00–7.01]	0.64 [0.07–5.67]	0.69	11	1 (2)	2.0	0.09 [0–0.41]	0.47 [0.07–3.05]	0.43
Month of infection													
August	33	6 (5)	909	97.36	0.66 [0.19–1.65]	1.00	ref	33	5 (6)	1.2	0.15 [0.05–0.32]	1.00	ref
September	78	9 (9)	2548	97.84	0.35 [0.16–0.67]	0.45 [0.12–1.78]	0.26	78	9 (9)	1.0	0.12 [0.05–0.21]	0.76 [0.28–2.10]	0.60
October	95	84 (23)	3986	93.15	2.11 [1.23–3.35]	1.92 [0.58–6.42]	0.29	149	24 (85)	3.5	0.16 [0.11–0.23]	1.06 [0.44–2.58]	0.89
November	151	74 (30)	4492	85.28	1.65 [1.03–2.50]	1.77 [0.55–5.75]	0.34	349	56 (150)	2.7	0.16 [0.12–0.20]	1.06 [0.46–2.46]	0.89
December	84	23 (14)	2656	78.43	0.87 [0.38–1.69]	1.11 [0.32–3.91]	0.87	175	36 (79)	2.2	0.21 [0.15–0.27]	1.36 [0.58–3.20]	0.49
**Total**	**441**	**196 (81)**	**14 591**	**89.09**	**1.34 [0.99**–**1.78]**	**n.a.**	**n.a.**	**784**	**130 (329)**	**2.5**	**0.17 [0.14**–**0.19]**	**n.a.**	**n.a.**

Left side of the table displays secondary attack rates (SARs), defined as the proportion of SARS-CoV-2-PCR positive secondary cases in a given cohort of close contact persons around that index case. Incidence rate ratios (IRRs) and corresponding confidence intervals (CIs) and *P*-values from negative binomial regression in 14 591 high-risk contacts clustered in cohorts around 441 index cases compare the mean count of secondary cases per index-cohort by the characteristic of index case, where an IRR of 1.0 corresponds to H_0_ = ‘there is no difference in the SAR between groups’. The analyses take account of the clustering of secondary cases within index cases. The right side of the table displays the cluster risk, i.e. the risk of causing at least one secondary infection among high-risk contacts, and associated risk ratios (RRs) from binomial regression for the comparison between groups, where an RR of 1.0 corresponds to H0 = ‘there is no difference in cluster risk between comparison groups’.

a Subgroup of index cases with complete information on a number of contact persons and a number of PCR tests.

b Complete study population, i.e. presents additional data on secondary cases around index cases with incomplete information on negative PCR tests in close contact persons, thus not allowing to calculate SARs.

c Presented data include teachers.

The SAR varied by the characteristic of the index case: role (teacher > student/child, IRR = 3.17, *P* < 0.001), symptom status at the time of diagnosis (pre-/asymptomatic < symptomatic, IRR = 0.47, *P* = 0.02), type of institution (day-care centres > secondary schools, IRR = 3.23, *P* < 0.001) and age (older than 35 years > age between 6 and 21 years) (Table 1, left).

### Cluster composition by role of index case and type of institution

Teacher-indexes were associated with on average more secondary cases (169/157, mean number of secondary cases per index (MSPI) = 1.08) than students/children (145/591, MSPI = 0.25; estimated MSPI ratio = 4.39, *P* < 0.001) (Table 2). Assessing transmission patterns by the role of index and secondary cases, we found that the average number of teacher-secondaries associated with student/child-indexes was 0.04 MSPI (corresponding to about one teacher secondary case in 25 student/child-index cases), compared to 0.56 teacher-secondaries associated with teacher-indexes (one teacher secondary case in two teacher-index cases, estimated MSPI ratio 13.25, *P* < 0.0001). A similar comparison looking at secondary cases in children/students found a similar but less pronounced difference towards student/child-secondaries being more likely identified in clusters around teacher-indexes (81/157, MSPI = 0.52) compared to those around students/children-indexes (120/591, MSPI = 0.20, estimated MSPI ratio 1.54, *P* < 0.001). Looking at role-specific cluster composition while stratifying by type of institution identified on average 1.26 MSPI around teacher-indexes in day-care, roughly evenly divided between teacher-secondaries (0.66 MSPI) and children-secondaries (0.59 MSPI). In schools, about 0.50 MSPI were identified around teacher-indexes, mostly in student-secondaries (0.44 MSPI), while teacher-secondaries were rare (0.06 MSPI). We observed on average two secondary cases per three child-indexes in day-care institutions (0.66 MSPI), which were about evenly divided between child-secondaries (0.38 MSPI) and teacher-secondaries (0.28 MSPI). Student-indexes in schools had the lowest MSPI: on average only one secondary per six student-indexes (0.17 MSPI). Among these, we hardly ever observed teacher-secondaries (0.004 MSPI).

**Table 2.** Cluster composition by type of educational institution and role of SARS-CoV-2 index case, Germany, 2020

Characteristic of index case		All types of secondary cases (*n* = 314)^a^				Student/child secondaries (*n* = 201)				Teacher secondaries (*n* = 113)			
		*N* cases (clusters)	MSPI^b^	MSPI ratio [95% CI]^c^	*P*-value^c^	*N* cases (clusters)	MSPI^b^	MSPI ratio [95% CI]^c^	*P*-value^c^	*N* cases (clusters)	MSPI^b^	MSPI ratio [95% CI]^c^	*P*-value^c^
All institutions	Student/child, *n* = 591	145 (76)	0.25	1.00		120 (70)	0.20	1.00		25 (16)	0.04	1.00	
	Teachers, *n* = 157	169 (48)	1.08	4.39 [2.67–7.21]	<0.001	81 (37)	0.52	2.54 [1.52–4.23]	<0.001	88 (32)	0.56	13.25 [6.59–26.67]	<0.001
Day-care	Student/child, *n* = 79^d^	52 (20)	0.66	1.00		30^d^ (15)	0.38	1.00		22^d^ (13)	0.28	1.00	
	Teachers, *n* = 113^d^	142 (38)	1.26	1.91 [0.95–3.84]	0.07	67^d^ (32)	0.59	1.56 [0.78–3.13]	0.21	75^d^ (26)	0.66	2.38 [1.04–5.49]	0.04
Schools	Student/child, *n* = 474^d^	82 (52)	0.17	1.00		80^d^ (51)	0.17	1.00		2^d^ (2)	0.004	1.00	
	Teachers, *n* = 32^d^	16 (6)	0.50	2.89 [0.99–8.44]	0.05	14^d^ (5)	0.44	2.59 [0.86–7.83]	0.09	2^d^ (2)	0.06	14.81 [2.09–105.15]	0.007

Table displays the mean number of SARS-CoV-2-PCR positive secondary cases per index case (MSPI), conditional on the role of the index case (i.e. student/child *vs.* teacher), and stratified by type of institution (day-care centre, school). For instance, independent of institution, the mean number of secondary cases in teachers is 0.04 and 0.56 for student/child and teacher-index cases, respectively, corresponding to an MSPI ratio of 13.3; the same comparison for day-care centres results in an MSPI ratio of 2.4.

a 36 index cases with missing age/role information.

b Mean number of secondary cases per index case (MSPI).

c MSPI ratio and corresponding confidence intervals (CIs) and *P*-values from negative binomial regression, modelling the difference in MSPI by category of index case, where an MSPI ratio of 1.0 corresponds to H_0_ = ‘no difference in the mean count of secondary cases between index categories’.

d Number of subjects do not add up to total due to exclusion of cases that attended institutions other than schools and day-care centres (e.g. special needs schools).

### Index case, cluster size and cluster-composition

**Fig. 2.**
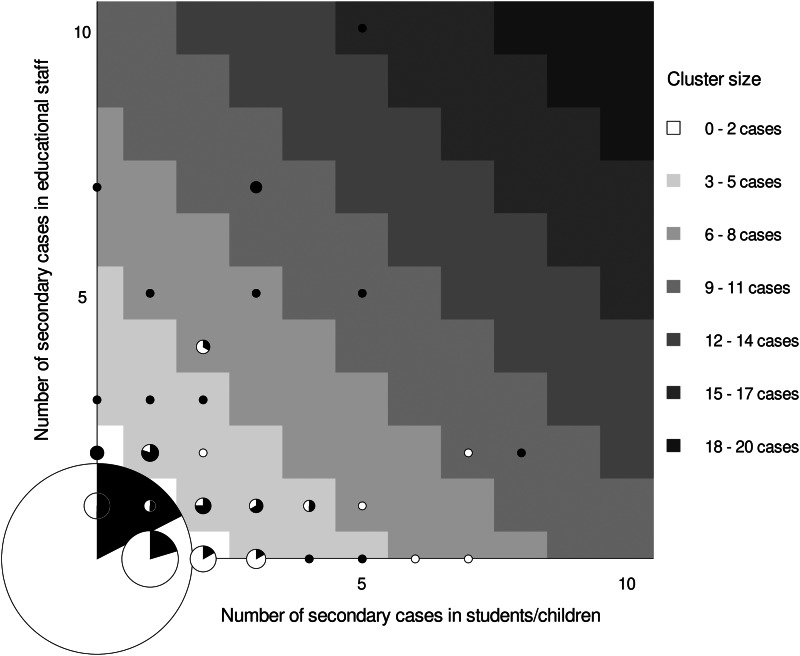
Frequency of secondary cases in children and teachers by the role of SARS-CoV-2-PCR positive index case. Graph displays frequency and role of 784 index cases and their association with secondary transmission to teachers and students/children in schools and day-care centres in Rhineland-Palatinate, Germany, 2020. Grid position of circles represents the number of secondary cases in students/children (*x*-axis) and teachers (*y*-axis). The circle size is proportional to the number of index cases with a particular number of secondary cases observed in this study. The colour inside the circle represents the share of children (white) and teachers (black) observed among index cases represented by that circle. Circles in areas of the grid with the same shade of grey represent clusters of similar size. For instance, the black dot at the very top of the grid identifies one cluster of size 15 (high cluster size = dark shade of grey) that emerged around a teacher index case (indicated by black colour *vs*. white colour of dot) and led to the identification of 10 secondary cases in teachers (position on *y*-axis) and 5 in student/children (position on *x*-axis).

The 329 secondary cases reported in this study occurred in 130 clusters, while the majority, 654 of overall 784 indexes (83%), were associated with zero secondary cases. In those 130 cases, where transmission occurred, the average cluster size was 2.5 secondary cases (Supplementary Fig. S2). There were nine clusters reported with seven or more secondary cases (Table 3), of which seven (78%) were associated with a teacher-index. Seven of the large outbreaks were in day-care centres for young children, where the index cases had on average 78 category-I contacts. All nine outbreaks occurred in settings, where the index cases had a large number of category-I contacts (between 37 and 166 contacts), as opposed to an average of 33 contacts per index case in the overall study. Larger outbreaks were more often associated with teacher- than child-/student-indexes (mean cluster size 3.5 *vs.* 1.9) explaining why ‘black dots’ prevail when moving from the lower left to the upper right corner of the grid in Figure 2. Outbreaks involving several teachers follow commonly on an index case in teachers and rarely on an index in children/students.

**Table 3.** Contextual information on the nine largest (size ≥7) among 130 clusters emerging from overall 784 SARS-CoV-2-PCR positive index cases in educational institutions, Germany, August to December 2020

	Month of symptom onset^a^	Role of index	Age group of index^b^	Type of institution	Number of high-risk contacts^c^		Number of secondary cases		
					Total	% PCR-tested	Total	Children	Teachers
Cluster 1	December	Teacher	41–45	Day-care ≤ 6 years	43	Unknown	8	3	5
Cluster 2	October	Child	0–5	Day-care ≤ 6 years	150	70%	9	7	2
Cluster 3	December	Teacher	41–45	Day-care ≤ 6 years	45	Unknown	10	5	5
Cluster 4	November	Teacher	41–45	Day-care ≤ 6 years	79	86%	10	8	2
Cluster 5	October	Teacher	21–25	Day-care ≤ 6 years	87	85%	10	3	7
Cluster 6	November	Teacher	16–20	Day-care ≤ 6 years	37	Unknown	10	3	7
Cluster 7	October	Teacher	51–55	Day-care ≤ 6 years	106	95%	15	5	10
Cluster 8	October	Child	11–15	Secondary school	166	100%	7	7	0
Cluster 9	November	Teacher	46–50	Unknown	56	Unknown	7	0	7

a Date of symptom onset for symptomatic cases, date of test for asymptomatic cases.

b In years.

c High risk is defined as a person who stayed face to face (<1.5 m) for 15 min or longer, or in the same room for 30 min or longer with a COVID-19 case, respectively [20]. In crowded or unclear situations, or when resources do not allow for an individual risk assessment, particularly in the context of schools and day-care centres, all members of a class or group may be classified as high-risk contact persons [17].

## Discussion

This study provides evidence for an overall low SARS-CoV-2 transmission risk in educational settings from August to December 2020 in Germany, a period with sharply increasing incidence rates, but with preventative measures in place. We found that approximately 1.3% of school contacts of an index case classified as category-I became SARS-CoV-2-positive. When restricting the denominator to PCR-tested contacts, we estimated a comparable SAR of 1.5%. These numbers are well in line with other published findings on the risk of transmission in schools from Australia, Germany, Italy, Ireland and Singapore, where several smaller studies found comparable SARs between 0% and 3% [4–8, 24].

Compared to student-/children-indexes, we found that the SAR was higher when the index case was a teacher. Likewise, the cluster risk and the mean number of secondary cases were higher when teachers were identified as index cases compared to students. Although not formally tested in other studies, mainly due to the small sample size, descriptive findings in published literature already point towards larger numbers of secondary cases in teacher-index cases and support our findings [5, 25–27]. In one study from the UK, half of 18 primary school outbreaks involved teachers only [25]. At the same time, we found that child-indexes were less likely to be associated with secondary infection in teachers and that outbreaks following child-indexes with secondary infection in teachers are more likely to occur in the day-care setting: in association with 591 student/child-indexes, 25 teachers were identified as secondary cases, of which 22 were observed in 13 outbreaks in day-care centres and only three teachers in three outbreaks in schools.

The role of asymptomatic cases in the spread of the COVID-19 pandemic has been a subject of an ongoing debate. A review and meta-analysis found that the proportion of asymptomatic cases was 17% of all COVID-19-positive cases [28, 29]. They further report that the risk of transmission was about 40% lower in asymptomatic cases as compared to symptomatic cases. While this is in line with our finding of a 50% lower SAR in contacts of asymptomatic cases, we would like to interpret this finding with caution. Indeed, what we observed as ‘asymptomatic’ may in some cases just have been a PCR-diagnosis in the ‘pre-symptomatic’ phase, e.g. in contact persons of COVID-19-positive cases outside the school/day-care setting. Hence, ‘asymptomatic’ cases in the presented study may in fact just have spent less days of their infectious period in school/day-care, thus explaining the lower risk of transmission. At the same time, these findings do not support the prevailing fear that asymptomatic cases could play a major role in the transmission of COVID-19 in schools/day-care under the current hygiene measures. One explanation for this finding, apart from shorter contact times due to ‘pre-symptomatic’ diagnosis, is a potential lower viral load in asymptomatic *vs.* symptomatic cases [30].

This study has limitations. First, we do not have follow-up data of all notified cases in the context of educational institutions from all 16 reporting DPHAs. This raises the question of selection bias. However, we advised DPHAs to report consecutive index cases over at least a 4-week period or longer, thus reducing the chance of systematic under- or over-reporting of more or less salient index cases and associated under- or overestimation of transmission risk. Second, although all DPHAs routinely offer PCR tests to all contact persons to a COVID-19 index case at high risk of transmission in the educational setting, 44% of our sample came from DPHAs that had outsourced sampling and testing to community testing centres. From these DPHAs, we received reliable information on secondary cases, but not on total contact persons and contact persons tested, since only positive test results are notifiable by testing centres and associated laboratories. However, considering the similarity of the average number of secondary cases and average cluster size in both samples 196/441 = 0.44 *vs.* 329/784 = 0.42 and 196/81 = 2.4 *vs.* 329/130 = 2.5, respectively (see Table 1), we are confident that index and secondary cases in both samples came from the same source population.

A third limitation concerns the quality of information that allows the DPHA to identify the underlying chain of transmission and thus to judge whether the index case was indeed the primary case in the given setting. Hypothesizing that some index cases were included although not being the primary case and knowing that children are more likely to have asymptomatic infection compared to adults, this may have led to an overestimation of the risk of transmission around teacher-secondaries through increasing the risk of misclassifying children as secondary cases. Although the presented risk and MSPI ratios may to a limited extent be an overestimation of the true difference between teachers and children/students, it seems unlikely that misclassification explains the whole magnitude of the difference observed in our data. This seems particularly true since the proportion of ‘entirely asymptomatic cases’, even in small children, is a function of how the investigation was performed and tends to be substantially higher in settings where superficial investigations are used [28, 29]. The in-depth investigations performed by the DPHAs for each of the presented 784 SARS-CoV-2 index cases will thus have greatly minimized this bias in our work.

Fourth, our results may be limited by representing the transmission situation in the educational setting at times, where literally no SARS-CoV-2 variants of concern have been present in Germany, yet. Although some of these have been shown to be associated with higher transmissibility rendering the absolute estimates presented outdated, our findings on transmission patterns and relative differences on transmission by setting and role are likely to be independent of strain type and thus to hold true in the presence of variants of concern as well.

Finally, our study attributes all transmissions detected around COVID-19 indexes in the educational setting to transmission in schools or day-care. This approach does not acknowledge that children/students and teachers may also have contact with each other outside the institution, e.g. during leisure activities. This may have increased the presented risk estimates, particularly for child-to-child transmission, where exposure in the classroom and during leisure commonly coincides.

## Conclusions

During a period of sharply increasing COVID-19 incidence rates in the population from August to December 2020, we found a low and rather stable transmission risk in schools, which provides evidence for the effectiveness of the preventative measures in place in German educational institutions. We found evidence for an increased risk of SARS-CoV-2 transmission to high-risk contact persons to teacher-indexes in day-care centres, mainly due to a stronger association of teacher-indexes with teacher-secondaries. Much less SARS-CoV-2 infection, by contrast, was found among high-risk contact person to children-/student-index cases and only a negligible number of infections could be observed when school-teachers were close contacts to student-indexes. The early communication of the presented findings informed public health decisions in Germany and Europe [31–33]. Continuous surveillance of SARS-CoV-2 transmission in the educational setting can powerfully inform public health policy and the public alike, and has proven to be an important tool to balance educational justice and anti-epidemic measures during the COVID-19 pandemic.

## Data Availability

Data and analytic code used for this study will be shared immediately upon request to the corresponding author.

## Supplementary material

For supplementary material accompanying this paper visit http://dx.doi.org/10.1017/S0950268821002077. click here to view supplementary material

## References

[1] Armitage R and Nellums LB (2020) Considering inequalities in the school closure response to COVID-19. The Lancet Global Health 8, e644. 32222161 10.1016/S2214-109X(20)30116-9 PMC7195275

[2] Ravens-Sieberer U (2020) Mental health and quality of life in children and adolescents during the COVID-19 pandemic – results of the COPSY study. Deutsches Ärzteblatt International 117, 828–829. 33568260 10.3238/arztebl.2020.0828 PMC8005842

[3] Esposito S and Principi N (2020) School closure during the coronavirus disease 2019 (COVID-19) pandemic: an effective intervention at the global level? JAMA Pediatrics 174, 921–922. 32401277 10.1001/jamapediatrics.2020.1892

[4] Macartney K (2020) Transmission of SARS-CoV-2 in Australian educational settings: a prospective cohort study. The Lancet Child & Adolescent Health 4, 807–816. 32758454 10.1016/S2352-4642(20)30251-0 PMC7398658

[5] Larosa E (2020) Secondary transmission of COVID-19 in preschool and school settings in northern Italy after their reopening in September 2020: a population-based study. Eurosurveillance 25, 2001911. 10.2807/1560-7917.ES.2020.25.49.2001911 PMC7730487 33303065

[6] Heavey L (2020) No evidence of secondary transmission of COVID-19 from children attending school in Ireland. Eurosurveillance 25, 2000903. 10.2807/1560-7917.ES.2020.25.21.2000903 PMC7268273 32489179

[7] Yung CF (2020) Novel coronavirus 2019 transmission risk in educational settings. Clinical Infectious Diseases 72, 1055–1058. 10.1093/cid/ciaa794 PMC7337629 32584975

[8] Heudorf U (2020) Keine Pandemie-Treiber (*not the drivers of the pandemic*). Deutsches Ärzteblatt 117, A 2505–A 2508.

[9] Brauner JM (2021) Inferring the effectiveness of government interventions against COVID-19. Science (New York, N.Y.) 371, eabd9338. 10.1126/science.abd9338 PMC7877495 33323424

[10] Persson J, Parie JF and Feuerriegel S (2021) Monitoring the COVID-19 epidemic with nationwide telecommunication data. Proceedings of the National Academy of Sciences of the USA 118, e2100664118. 34162708 10.1073/pnas.2100664118 PMC8256040

[11] Iwata K, Doi A and Miyakoshi C (2020) Was school closure effective in mitigating coronavirus disease 2019 (COVID-19)? Time series analysis using Bayesian inference. International Journal of Infectious Diseases: IJID 99, 57–61. 32745628 10.1016/j.ijid.2020.07.052 PMC7836901

[12] Banholzer N (2021) Estimating the effects of non-pharmaceutical interventions on the number of new infections with COVID-19 during the first epidemic wave. PLoS ONE 16, e0252827. 34077448 10.1371/journal.pone.0252827 PMC8171941

[13] Thompson HA (2021) SARS-CoV-2 setting-specific transmission rates: a systematic review and meta-analysis. Clinical Infectious Diseases 73, e754–e764. 33560412 10.1093/cid/ciab100 PMC7929012

[14] Statistisches Landesamt Rheinland-Pfalz (2020) Kinder- und Jugendhilfe – Teil III.1/Teil III.3. Kinder und tätige Personen in Tageseinrichtungen und in öffentlich geförderter Kindertagespflege (*child and youth welfare – part III.*1*/part III.*3. *Children and staff in day-care institutions and government-financed child care*). Available at https://www.statistik.rlp.de/fileadmin/dokumente/berichte/K/1073/K1073_201900_1j_K.pdf (Accessed 18 Jan 2021).

[15] Statistisches Landesamt Rheinland-Pfalz (2020) Allgemeinbildende Schulen im Schuljahr 2019/2020. Teil 1: Schülerinnen und Schüler, Schulabgängerinnen und Schulabgänger. Available at http://www.statistik.rlp.de/fileadmin/dokumente/berichte/B/1013/B1013_201900_1j_K_T1.pdf (Accessed 18 Jan 2021).

[16] Ministerium für Bildung R-P (2020) Lüften und Raumlufthygiene in Schulen in Rheinland-Pfalz. Ergänzende Hinweise zum Hygieneplan-Corona für Schulen (*Ventilation and room air hygiene in schools in Rhineland-Palatinate. Complementary advice on the Corona-hygiene plan for schools*). https://corona.rlp.de/fileadmin/bm/Bildung/Corona/Handreichung_Lueften_und_Raumlufthygiene.pdf (Accessed 18 Jan 2021).

[17] Robert Koch Institut (2020) Präventionsmaßnahmen in Schulen während der COVID-19-Pandemie. Empfehlungen des Robert Koch-Instituts für Schulen (*Prevention measures in schools during the COVID*-19*–pandemic. Recommendations for schools by the Robert Koch-Institute*), 12.10.2020. Available at https://www.rki.de/DE/Content/InfAZ/N/Neuartiges_Coronavirus/Praevention-Schulen.pdf;jsessionid=F24221B6E926F39BFD127C5DBB7A7870.internet071?__blob=publicationFile (Accessed 21 Dec 2020).

[18] Ministerium für Bildung R-P (2020) Hygieneplan-Corona für die Schulen in Rheinland-Pfalz (*Corona hygiene plan for schools in Rhineland-Palatinate*). Available at https://corona.rlp.de/fileadmin/bm/Bildung/Corona/20201203_6._Hygieneplan_Corona_Schulen.pdf (Accessed 18 Jan 2021).

[19] Gewerkschaft Erziehung und Wissenschaft Rheinland-Pfalz (2020) Gemeinsame Empfehlungen des Ministeriums für Bildung, der Kommunalen Spitzen und des Landesamtes für Soziales, Jugend und Versorgung zur Anpassung der Hygienepläne der Kindertageseinrichtungen in Rheinland-Pfalz betreffend ‘Corona’ (*Consolidated recommendations for the adaptation of hygiene plans in day-care institutions for children in Rhineland-Palatinate in relation to ‘Corona’*). Available at https://www.gew-rlp.de/index.php?eID=dumpFile&t=f&f=96634&token=5229e8b7c87606e61daca0675fa9f89620124d4a&sdownload=&n=2020-04-29-Empfehlungen-Hygiene-Kita-.pdf (Accessed 03 Mar 2021).

[20] Robert Koch Institut (2020) Kontaktpersonennachverfolgung bei SARS-CoV-2-Infektionen (*Contact tracing of SARS-CoV-*2*-Infections*): Robert Koch Institut, 17.12.2020. Available at https://www.rki.de/DE/Content/InfAZ/N/Neuartiges_Coronavirus/Kontaktperson/Grafik_Kontakt_allg.pdf?__blob=publicationFile (Accessed Dec 21 2020).

[21] Clopper CJ and Pearson ES (1934) The use of confidence or fiducial limits illustrated in the case of the binomial. Biometrika 26, 404–413.

[22] StataCorp (2019) Stata Statistical Software: Release 16. College Station, TX: StataCorp LLC.

[23] SAS (2013). Statistics Analysis Systems, SAS Institute Inc. Cary, NC, USA.

[24] Xu W (2020) What is the evidence for transmission of COVID-19 by children in schools? A living systematic review. Journal of Global Health 10, 021104. 33437465 10.7189/jogh.10.021104 PMC7774027

[25] Ismail SA (2021) SARS-CoV-2 infection and transmission in educational settings: a prospective, cross-sectional analysis of infection clusters and outbreaks in England. The Lancet Infectious Diseases 21, 344–353. 33306981 10.1016/S1473-3099(20)30882-3 PMC7833602

[26] Ehrhardt J (2020) Transmission of SARS-CoV-2 in children aged 0 to 19 years in childcare facilities and schools after their reopening in May 2020, Baden-Württemberg, Germany. Eurosurveillance 25, 2001587. 10.2807/1560-7917.ES.2020.25.36.2001587 PMC7502898 32914746

[27] Gold JAW (2021) Clusters of SARS-CoV-2 infection among elementary school educators and students in one school district – Georgia, December 2020–January 2021. Morbidity and Mortality Weekly Report (MMWR) 70, 289–292. 33630823 10.15585/mmwr.mm7008e4 PMC8344983

[28] Byambasuren O (2020) Estimating the extent of asymptomatic COVID-19 and its potential for community transmission: systematic review and meta-analysis. Official Journal of the Association of Medical Microbiology and Infectious Disease Canada 5, 223–234. 10.3138/jammi-2020-0030 PMC9602871 36340059

[29] Vermund SH and Pitzer VE (2021) Asymptomatic transmission and the infection fatality risk for COVID-19: implications for school reopening. Clinical Infectious Diseases 72, 1493–1496. 32584967 10.1093/cid/ciaa855 PMC7337644

[30] Zhou R (2020) Viral dynamics in asymptomatic patients with COVID-19. International Journal of Infectious Diseases 96, 288–290. 32437933 10.1016/j.ijid.2020.05.030 PMC7211726

[31] Robert Koch Institute (2021) Epidemiologisches Bulletin 23/2021. STIKO: 6. Aktualisierung der COVID-19-Impfempfehlung Empfehlung bei Lieferengpässen von Impfstoffen (*Update of the COVID-*19 *vaccination recommendation recommandation in case of delivery shortages of vaccines*). https://www.rki.de/DE/Content/Infekt/EpidBull/Archiv/2021/Ausgaben/23_21.pdf?__blob=publicationFile (Accessed 17 Jun 2021).

[32] BARNE-U-OF (2021) Statusrapport 12. Utsatte barn og unges tjenestetilbud under covid-19–pandemien (*Vulnerable children and young people's services during the COVID-*19 *pandemic*). Available at https://www.regjeringen.no/contentassets/07a94a46945c43408c50a168e540079d/statusrapport-nr-12-fra-koordineringsgruppen-til-bfd.pdf (Accessed 17 Jun 2021).

[33] European Centre for Disease Prevention and Control (2021) TECHNICAL REPORT. COVID-19 in children and the role of school settings in transmission - second update. Available at https://www.ecdc.europa.eu/sites/default/files/documents/COVID-19-in-children-and-the-role-of-school-settings-in-transmission-second-update.pdf (Accessed 04 Aug 2021).

