# Supplementary material for: Surveillance of SARS-CoV-2 transmission in educational institutions, August to December 2020, Germany
Source: Epidemiol Infect. 2021 Sep 22;149:e213. doi: 10.1017/S0950268821002077 (PMC8503068; doi:10.1017/S0950268821002077)

Supplementary Material to Schoeps A, et al. Surveillance of COVID-19 transmission in educational institutions, August to December 2020, Germany

Supplementary Figure 1: Time point of PCR testing in 12,804 contact persons to 426 SARS-CoV-2 index cases in educational institutions, Rhineland-Palatinate, 2020

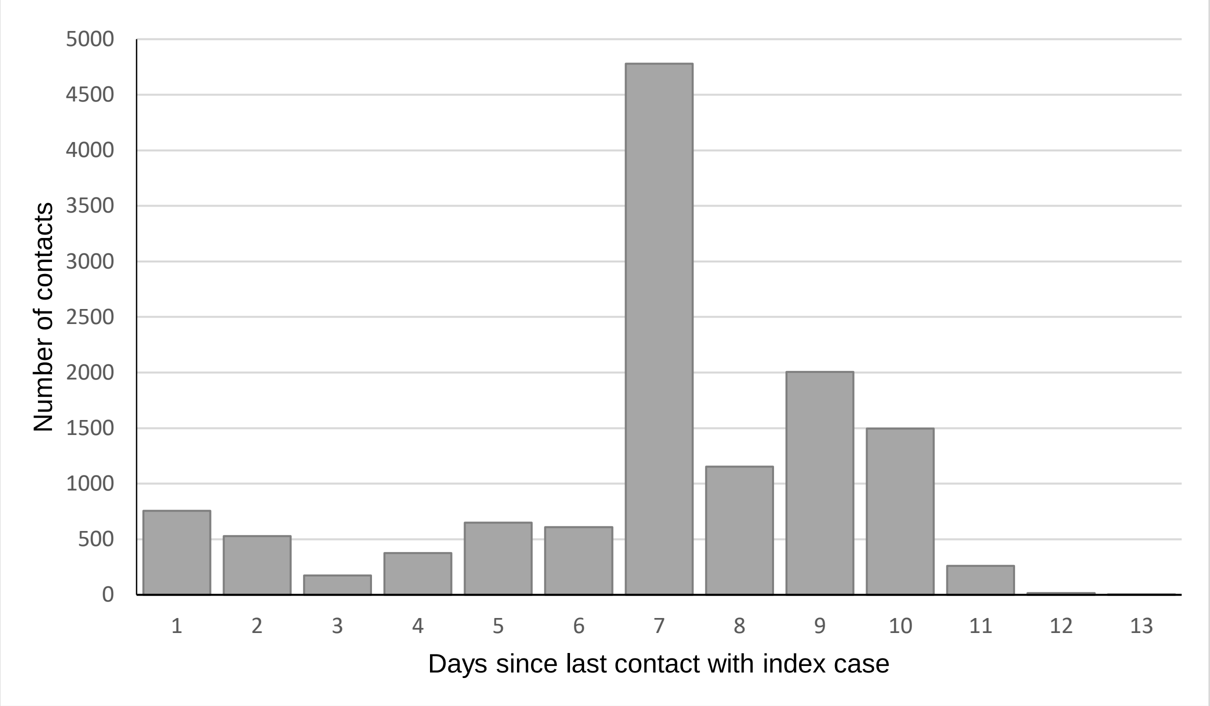

Supplementary Figure 2: Frequency of custer sizes (log-scale) around 784 SARS-CoV-2 index cases in educational institutions, Rhineland-Palatinate, 2020

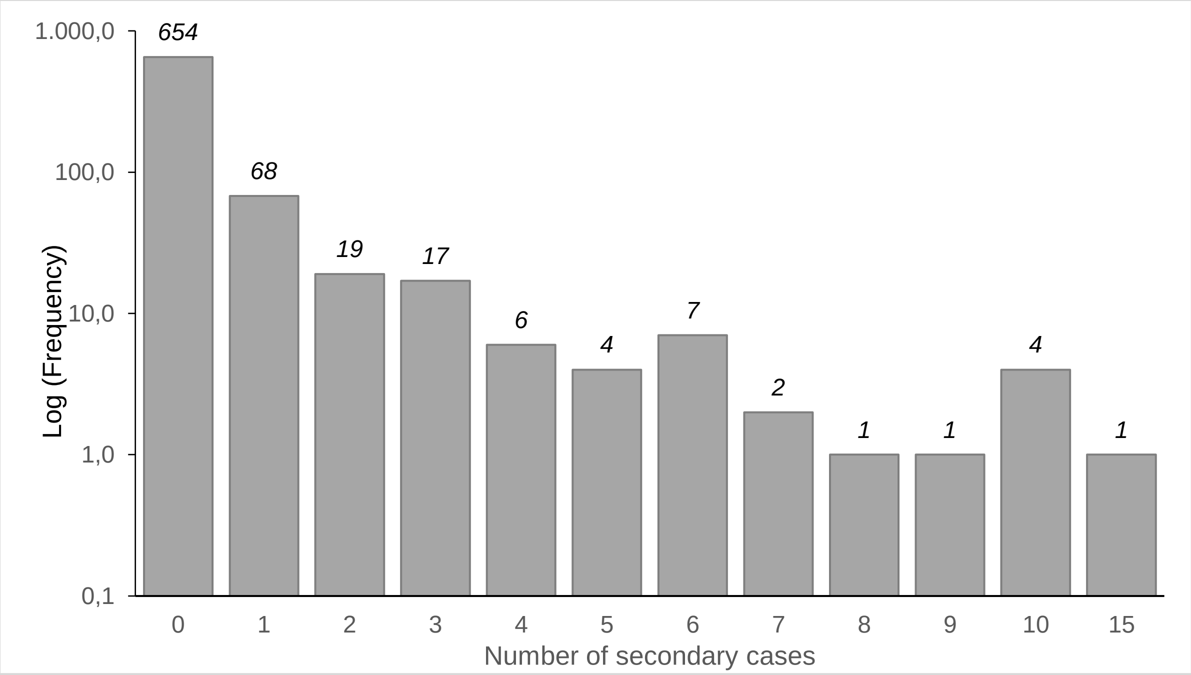

Supplement: Supplementary file 1 [file S0950268821002077sup001.pdf]
